# Artificial intelligence-based approaches for the detection and prioritization of genomic mutations in congenital surgical diseases

**DOI:** 10.3389/fped.2023.1203289

**Published:** 2023-08-01

**Authors:** Qiongfen Lin, Paul Kwong-Hang Tam, Clara Sze-Man Tang

**Affiliations:** ^1^ Department of Surgery, Li Ka Shing Faculty of Medicine, The University of Hong Kong, Hong Kong SAR, China; ^2^ Faculty of Medicine, Macau University of Science and Technology, Macau, Macau SAR, China; ^3^ Dr Li Dak-Sum Research Centree, The University of Hong Kong - Karolinska Institutet Collaboration in Regenerative Medicine, Hong Kong, Hong Kong SAR, China

**Keywords:** artificial intelligence, congenital surgical diseases, variant detection, variant prioritization, bioinformatics

## Abstract

Genetic mutations are critical factors leading to congenital surgical diseases and can be identified through genomic analysis. Early and accurate identification of genetic mutations underlying these conditions is vital for clinical diagnosis and effective treatment. In recent years, artificial intelligence (AI) has been widely applied for analyzing genomic data in various clinical settings, including congenital surgical diseases. This review paper summarizes current state-of-the-art AI-based approaches used in genomic analysis and highlighted some successful applications that deepen our understanding of the etiology of several congenital surgical diseases. We focus on the AI methods designed for the detection of different variant types and the prioritization of deleterious variants located in different genomic regions, aiming to uncover susceptibility genomic mutations contributed to congenital surgical disorders.

## 1. Introduction

Congenital disorders, also known as congenital abnormalities or disabilities, are the leading causes of infant morbidity and mortality. Congenital surgical diseases refer to those medical conditions present at birth that require surgical intervention as the first-line treatment. Myriad factors, including genetic mutations, chromosomal abnormalities, and environmental factors such as toxins or virus infection can cause these conditions. Many of these congenital surgical diseases have been shown to have a strong genetic basis. For example, approximately 10%–30% of patients with congenital heart disease (CHD), the most common congenital anomaly that affects around 1% of newborns, may have an identified genetic cause (1, 2). Chromosomal anomalies (e.g., trisomy 21 and 22q11.2 deletion) and mutations in genes such as *GATA4*, *NOTCH1*, *NKX2-5* and *TBX1* dysregulating cardiac morphogenesis and differentiation have been identified in individuals with CHD (2). In addition, common regulatory variants and rare mutations also predispose to an increased risk of less common surgical disorders, such as Hirschsprung disease and biliary atresia (3).

In the past decade, the advancement in next-generation sequencing (NGS) has revolutionized precision medicine, shifting the paradigm of genetic diagnosis toward big data analytics. Now, researchers are able to elucidate the genetic etiology of congenital diseases by analyzing massive omics data generated from DNA, RNA and epigenetic sequencing. Although genomic analysis has been confirmed to be a potent approach for identifying disease-causal variants, the detection and prioritization of these variants predisposed to diseases from a mass amount of data is still a barrier for researchers to tackle with. AI affords from the tremendous amount of data remains challenging. AI fills in this research gap by offering compelling solutions to big data genomic analysis in three major aspects: (i) detection of high-confidence genomic mutations from various genomic data; (ii) predicting the functional impact of these variants on protein structure or functions or regulatory elements; and (iii) prioritizing disease-causing variants in patients.

AI is a technic acted by machines to mimic human intelligence. In computer science, AI is defined as the study of “intelligent agents”. It can deal with complicated problems by intelligently searching through different relevant datasets, excavating the hidden patterns of the existing features, formulating prediction models, and giving the best solution (4).

**Figure 1:**
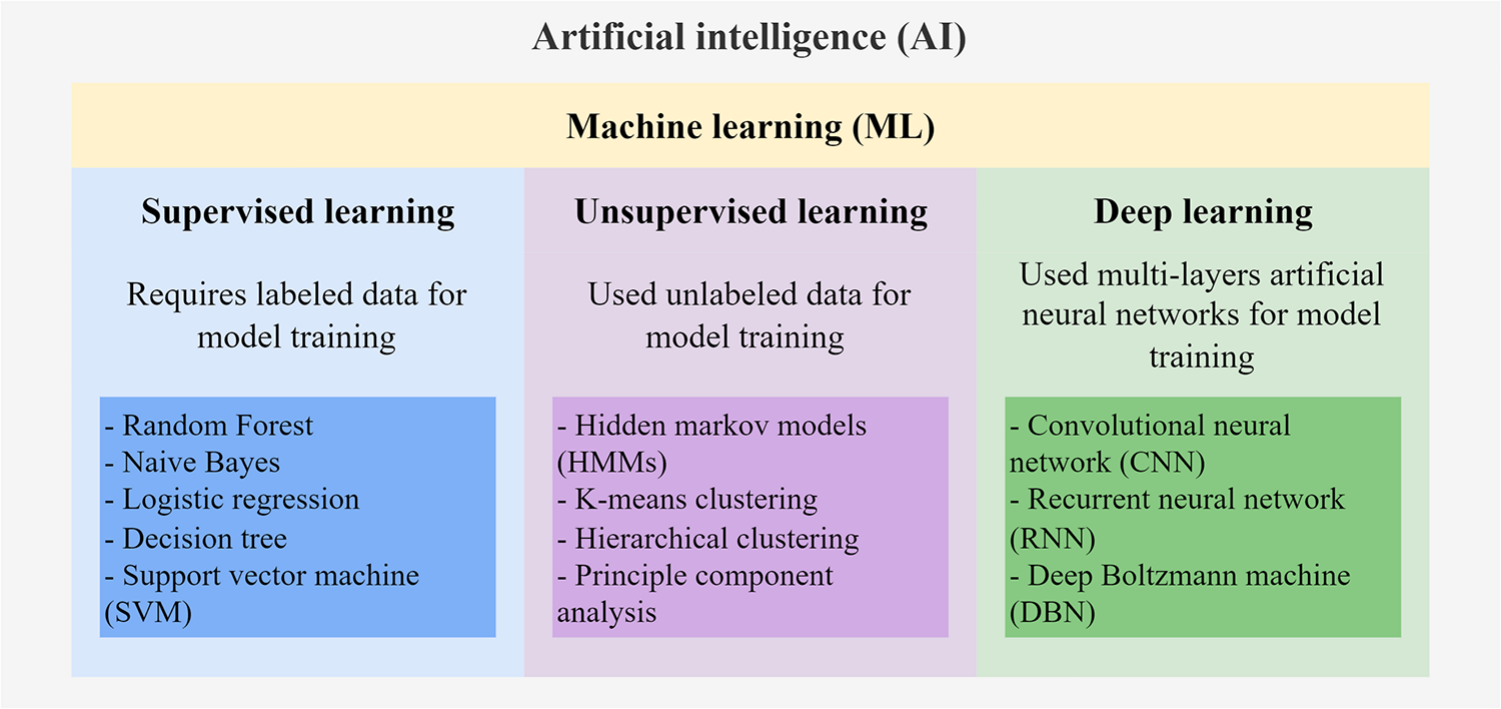
Schematic diagram of AI algorithms.

There are multiple subfields of AI, including machine learning (ML), deep learning (DL), and natural language processing (Figure 1). ML serves to build AI-driven applications using supervised or unsupervised learning methods (5, 6). Supervised learning uses labeled training data to train the ML model by learning the patterns and relationships between the input features. The trained model is then used to predict the labels of the new unlabeled testing data. Supervised learning algorithms, including Random Forest, Naïve Bayes, and Support Vector Machines (SVM), are mostly classification-and regression-based. The classification algorithm finds functions that help categorize the data into classes based on the input labels and is mostly applicable for binary or categorical data with discrete values. The regression algorithm predicts output labels based on the association between dependent and independent variables and is mostly used for predicting continuous data. On the other hand, unsupervised learning trains models with unlabeled data to explore hidden patterns in the input. Clustering and dimensionality reduction are common techniques for unsupervised learning methods, such as Hidden Markov models (HMMs) and k-means clustering. DL is the emerging machine learning subfield that trains models with massive data and various complex supervised or unsupervised algorithms. DL involves the use of multi-layer artificial neural networks to learn the complicated structures and patterns in the data. Among the DL algorithms, convolutional neural networks (CNN) and recurrent neural networks (RNN) are the most frequently used (7, 8). For comprehensive information on the details of these ML algorithms, interested readers can refer to other reviews (5, 6) that extend beyond the scope of genomic data applications discussed here.

With the introduction of these advanced AI-based, especially DL-based, detection and prediction tools, countless disease-causing variants were identified and prioritized from big genomic data, dramatically enhancing our understanding of the etiology of numerous congenital surgical diseases and promoting the uptake of this new evidence into clinical practice. In this review, we will focus on how AI assists the genomic analysis of congenital surgical diseases by improving the performance of detecting and prioritizing candidate disease-causing mutations.

## 2. Application of AI models in the identification of genetic variants

Variant calling is the critical process to detect genetic variations from DNA sequencing data. Before this process, the alignment of sequencing reads to the reference genome is required, then genetic variants are detected by comparing the differences in base sequence between the aligned reads and the reference genome. To detect high-quality genomic variants sensitively and specifically, numerous tools have been developed using different AI models (Bayesian, Random Forest, CNN, etc.). The majority of these tools are established to identify single-nucleotide variations (SNVs), small insertions and deletions (indels), and copy number variants (CNVs), as these types of variation are the dominant sources of a genomic mutation linked to disease. Similarly, rare and novel CNVs can also be called from traditional SNP (single-nucleotide polymorphisms) array data using AI models trained on data with known copy number information.

### 2.1. Detection of SNVs and indels

As the major variant types, SNVs and indels could be detected by plenty of variant calling tools, among which Genome Analysis Toolkit (GATK), DeepVariants, and FreeBayes are frequently used. GATK is the most widely used programming framework for analyzing DNA sequencing data and for the discovery of SNVs and indels. It applies various machine learning methods like logistic regression, HMM, and Naïve Bayes classification to reduce base errors and capture high-quality variants. For example, in the variant quality score recalibration (VQSR) process to filter low-quality variants, GATK trained on multiple variant annotations (e.g., genotype qualities, depth of coverage, mapping qualities, and local sequence context, etc.) of high-confident known variants like HapMap genotypes and Omni 2.5 genotypes for 1,000 Genomes samples (9). It then uses the trained model to assign a well-calibrated variant quality score to each variant in a callset and refines the callset to a desired high level of truth sensitivity (10–12).

Unlike GATK, another deep learning-based variant caller called DeepVariants accurately identifies genetic variants using a single deep CNN model trained with known genotypes instead of the combination of multiple statistical models. Using the Inception architecture, the CNN model calculates the genotype likelihoods for each site using a pileup image of the reference genome and sequenced reads around each candidate variant (13). FreeBayes, a Bayesian variant calling tool, uses a haplotype-based method to read short haplotypes directly from sequencing data. It offers many advantages for variants detection compared to approaches that manipulate a single site simultaneously. To maintain semantic consistency between the candidate variants, the haplotype-based method will assess all categories of alleles in the same sequencing context simultaneously, improving the detection utilities and accuracy (14).

### 2.2. Detection of CNVs

Another variant type, CNVs, is the variant that exhibits differences in the number of copies in specific DNA segments, specifically manifested as duplication or deletion of a particular size of DNA fragments (15). A popular CNV caller, PennCNV, uses HMM algorithm to detect CNVs from intensity data generated by high-resolution SNP arrays. The parameters of HMM models were first optimized by training using the Baum-Welch algorithm on large CNV regions from a large set of training samples. The optimized HMM then models the observed intensity data as a mixture of normal distributions, incorporated with the Log R Ratio (LRR) and B Allele Frequency (BAF) values for each SNP in the genome to predicted copy number states (e.g., 0, 1, 2, or more copies) at each genomic location. Next, the detected CNVs will be validated using the posterior probabilities of each copy number state calculated by the Bayesian algorithm with the use of pedigree information to obtain reliable CNVs (16). The same framework was further extended and adapted for calling CNVs from whole genome sequencing (WGS) data in PennCNV-Seq. Another DL-based tool, DeepCNV, aims for CNV validation instead of CNV calling. It attempts to replace human visual examination in order to reduce the false positive rate of CNVs, centering around the CNVs called by PennCNV. DeepCNV is constructed by a hybrid deep neural network architecture consisting of a deep convolutional neural network (CNN) and a deep fully connected neural network (DNN). It can deal with both image data and summary statistics output from PennCNV, using CNN and DNN algorithms respectively. This tool has completely changed the ability of CNV studies and can trim the raw CNV calls into reliable CNV sets with high effectiveness and efficiency (17).

In the new era of high-throughput technology, various tools emerged for identifying CNVs from NGS data using AI models. Based on a machine-learning approach, CN-Learn accurately detects high-confidence CNVs by aggregating multiple CNV-detected methods (CANOES, CODEX, CLAMMS, and XHMM) from exome sequencing data. Caller-specific and genomic features such as GC content, CNV concordance, and CNV size were obtained from multiple CNV callers and further used as the training dataset for a Random Forest classifier, eventually used to distinguish true or false positive calls for the identified CNVs (18). Similarly, CNV-JACG is developed with a random forest model for Judging the Accuracy of CNVs and Genotyping using paired-end WGS data. CNV-JACG is trained on 21 distinct features characterizing true CNV regions, including 13 features characterizing the breakpoints of CNVs, 6 features of the region encompassed by the CNV, and 2 features related to the variants called within the CNV region. After training, the model learns to determine true and false CNVs and make predictions on the input dataset, calling real CNVs (19).

CNVs have been reported to have a high impact on congenital surgical diseases. For example, it has been reported that around 3%–25% of the CHD cases harbored rare pathogenic CNVs that could produce unproperly working proteins (2). To access the contribution of *de novo* CNVs in the pathogenesis of sporadic CHD, Glessner, J. T. et al. applied PennCNV and XHMM (exome hidden Markov model) for the detection of high-confident *de novo* CNVs from the genotyping array and whole exome sequencing (WES) data respectively (20). CNVs detected *in silico* were then validated experimentally using digital droplet PCR. Ultimately, they confirmed a significant increase in CNV burden in CHD cases compared with healthy controls (21).

Tetralogy of Fallot (TOF) is the most common subtype of CHD, characterized by pulmonary stenosis, ventricular septal defect, overriding aorta and hypertrophy of the right ventricle (22, 23). A WGS study on 146 Chinese nonsyndromic TOF parent-offspring trios CNV-JACG for the identification of high-confidence CNVs (>50 bp) from the WGS data. The study identified 16 *de novo* CNVs in 14 TOF patients, accounting for 9.6% in the Chinese TOF cohort, which is higher than that in the general population (24). CNV analysis on Hirschsprung disease (HSCR), also known as congenital intestinal aganglionosis, identified a novel candidate gene, NRG3, with an increased burden of intronic CNVs (both deletions and duplications) in patients. Furthermore, the CNV analysis also revealed the differential genetic architecture in relation to CNVs, such that syndromic HSCR was associated with longer CNVs whereas isolated HSCR were found to have an increased burden of shorter CNVs (25).

Biliary atresia (BA) is a rare pediatric hepatobiliary disorder with multifactorial etiology. It is characterized by progressive fibro-inflammatory obstruction of the bile duct. The exact cause of BA is still unknown, but it is thought to be caused by both genetic and environmental factors. Cheng et al. detected 29 BA-private CNVs from SNP array data of BA patients and controls using PennCNV, Birdseye and iPattern. By exploring the interconnectivity of CNVs, SNPs and genetic networks in BA patients, they observed a significant enrichment in the immune-inflammatory pathway for genes associated with these BA-associated CNVs (26).

## 3. Application of AI models in variant prioritization

Generally, the critical process of genomic analysis includes variant detection and variant annotation. Variants could be annotated with multiple variant features, like their associated gene symbol, protein consequence of nucleotide change, allele frequency, etc., among which deleterious prediction is the important term. With the predicted deleterious score, one could easily prioritize potentially damaging causative variants, facilitating the clinical interpretation of variants and thus contributing significantly to the study of congenital diseases.

### 3.1. Prioritizing deleterious mutations in the coding region

Combined Annotation-Dependent Depletion (CADD) is the most widely used annotation tool to predict the deleteriousness of short variants (SNVs and indels) in genetic studies of both monogenic and complex diseases. It applies a machine learning model to aggregate diverse annotations, including evolutionary conservation metrics from other annotated tools (phastCons scores, GERP, and phyloP), regulatory information and functional prediction, into a single, comprehensive measure, including evolutionary conservation, regulatory information, functional prediction score for each variant. Using the SVM algorithm, the model is trained on a set of known pathogenic and benign variants, learning to discriminate between these two classes based on the input annotations with high precision and accuracy for all kinds of variants like missense, splice, and frameshift variants (27, 28).

In contrast, another tool Rare Exome Variant Ensemble Learner (REVEL), is designed only to predict the pathogenicity of missense variants. Similar to CADD, REVEL is an ensemble method integrated with 13 other prediction tools: MutPred, FATHMM, VEST, PolyPhen, SIFT, PROVEAN, MutationAssessor, MutationTaster, LRT, GERP, SiPhy, phyloP, and phastCons. It is trained by Random Forest using a dataset of known pathogenic and rare neutral missense variants to predict the potential effect of the query variants. As reported, REVEL has better performance on pathogenicity prediction of missense variants than other ensemble methods: MetaSVM, MetaLR, KGGSeq, Condel, CADD, DANN, and Eigen and thus widely adopted for predicting *in silico* damaging effect (PP3) as supporting evidence of pathogenicity in ClinGen Expect specifications in variant interpretation (e.g., hearing loss Familial Hypercholesterolemia) (29–31).

AI-based variant annotation has been instrumental in the genetic analysis of rare congenital surgical diseases. In a WGS study of a Chinese cohort with TOF, Tang et al. extracted potential rare damaging variants by the damaging Phred-scaled CADD scores; thereby identified 6 TOF patients with ultra-rare damaging variants in 3 known TOF genes (*KDR*, *FLT4* and *NOTCH1*). It also pointed out novel biological pathways and developmental hotspots relevant to the dysregulation of cardiac development in TOF through enrichment analysis (24). Page et al. called variants using GATK and defined likely pathogenic nonsynonymous variants with a scaled CADD score ≥ 20, highlighting the increased burden of *NOTCH1* mutations in TOF (32). Likewise, a trio-based WES study on BA identified rare, deleterious *de novo* or biallelic variants in liver-expressed ciliary genes in 31.5% (28/89) of the BA patients with the help of the CADD, SIFT and PolyPhen2. They found that these rare deleterious variants in liver-expressed ciliary genes were associated with a significant two-fold increased risk of BA, underlying the potential disease mechanism of BA led by the malformation and dysfunction of cilia (33).

### 3.2. Prioritizing variants that may lead to alternative splicing

Alternative splicing is regulated by an extensive protein-RNA interaction network involving cis-elements within the pre-mRNA and trans-acting factors that bind to these cis-elements. It is a crucial regulator of gene expression, with around 15% of disease-causal mutations predicted to alter mRNA splicing (34). Disruption of splicing (for example, exon skipping and intron retention) would result in aberrant proteins that don't work correctly. Nowadays, numerous tools have been developed to predict the effects of splice variants, emphasizing whether variants in the splice regions can potentially lead to the loss or gain of the splice donor or splice acceptor.

SpliceAI uses an ultra-deep CNN model to computationally predict the effects of genetic variants on splicing based on the sequence of the pre-mRNA transcript. SpliceAI trained on the dataset from GENCODE (an integrated annotation of gene features) and the RNA-seq data Genotype-Tissue Expression (GTEx). Training on the GTEx RNA-seq dataset conduces to enhance the sensitivity of splicing-altering variation detection, particularly for detecting deep intronic splicing variants. Given a genetic variation, SpliceAI generates a couple of scores for the effects on acceptor/donor gain and acceptor/donor gain (35). Similar to SpliceAI, MMSplice (modular modeling of splicing) is a neural network-based model to predict the effects of variants on exon skipping, splice site choice, splicing efficiency, and pathogenicity. It consists of six modules scoring sequences from different genomic regions, wherein the donor and acceptor modules are trained using GENCODE annotation features, while the exon modules (exon 5’ and exon 3’ modules) and intron modules (intron 5’ and intron 3’ modules) are trained using massively parallel reporter assays (MPRAs) experiment, based on different module architectures. These six modules are combined with a linear model to score the variant effects on exon skipping, alternative donor/acceptor site, and splicing efficiency separately. Furthermore, it integrated with a logistic regression model to predict variant pathogenicity. For each input variant, MMSplice would output several scores, including (1) a main score that exhibits the effect of the variant on the inclusion level, (2) a pathogenicity score that shows the potential pathogenic effect, (3) an efficiency score that demonstrates the variant effect on splicing efficiency of the exon and (4) several scores for the effects of the acceptor/donor/exon/intron according to the reference allele and alternative allele (36).

In the context of genomic analyses, tools like SpliceAI and MMSplice are typically employed not in isolation but as part of a more extensive set of methods to prioritize pathogenic variants with deleterious effects. Belbin et al. explored a cryptic splice variant in *ABCB4*, predicted to cause a splice acceptor loss by SpliceAI (score = 0.39) through an IBD-based (identity-by-descent) phenome-wide association study (PheWAS) analysis and fine-mapping. It was further validated to disrupt the splicing of the ABCB4 pre-mRNA *in vitro*, leading to the skip transcription of exon 23, thus resulting in liver disease (37). Given the complex genetic architecture of the congenital disease, most of the time, researchers may not only employ tools for the prediction of coding variants but also adopt other tools for the prediction of splicing variants or regulatory variants. For example, a study that concentrated on the detection of mosaic mutation implicated in CHD captured deleterious missense variant by REVEL (with a score > 0.5) and damaging splicing variants by SpliceAI (with a delta score > 0.5) (38). Therefore, researchers would annotate the splice variants together with other variants using some ensemble tools or databases. Take CADD-Splice (same as CADD v1.6) as an example, it integrated with several superior ML-based methods (including SpliceAI and MMSplice) to score the potential splicing effect led by the genetic variations (39). On the other hand, dbNSFP is a comprehensive database designed to annotate the functional impact of all SNPs in the human genome. It complied dozens of prediction scores from various tools, consisting of (i) functional prediction (from SIFT, Polyphen, CADD, etc.), (ii) conservation scores (from phyloP, phastCons, GERP++, etc.), and (iii) many other variant annotations like allele frequency, gene information, protein information, splicing effect, regulatory elements, and gene-associated phenotype of mouse and zebrafish (40).

### 3.3. Prioritizing potentially damaging regulatory variants

Historically, the majority of diseases’ pathogenic variants are detected in the protein-coding regions, although it only takes up around 2% of human genomes. Nonetheless, disease-causing variations in the coding areas could only elucidate about 20%–50% of the diseases’ etiology, indicating that rare noncoding variations may contribute substantially to disease risk (41). Unlike coding variants that may affect protein structure, function and folding, noncoding variants disrupting functional regulatory elements (e.g., enhancers, insulators, promoters, etc.) have the potential to dysregulate gene expression and thus contribute to genetic diseases (42). Deleteriousness prediction tools primarily trained with coding datasets, like CADD and REVEL, are insufficient to predict the pathogenicity of noncoding variation. Hence, other variation annotation tools specialized in predicting the regulatory effect of noncoding variants are needed.

DeepSEA is a deep learning model specialized in predicting the functional effects of noncoding mutations. It uses a multi-layer CNN architecture to decode the regulatory sequence from massive epigenomic profiles and predict the chromatin effects of the genomic mutations. DeepSEA takes a 1,000 base pairs (bp) DNA sequence centered on each variant as input and creates a couple of sequences harboring either the reference or alternative allele at the variant position. Then it calculates the chromatin effect size across each epigenomic feature for each reference and alternative allele, in which the absolute differences between wild-type and mutation could be obtained. Additionally, DeepSEA also takes evolutionary conservation into account and computes the conservation score for each variant using PhastCons, PhyloP and GERP++. By incorporating the variant-phenotype information on human pathogenic variants from the Human Gene Mutation Database (HGMD), DeepSEA has the capacity to forecast the deleterious regulatory impacts that regulatory variations may have, thereby aiding in the prioritization of functional variations (43).

DeepSEA is a general deep learning model to predict the regulatory effects of noncoding variants for all kinds of diseases. HeartENN, on the other hand, is a heart-specific neural network built on top of DeepSEA to predict the epigenomic outcomes of variants in relation to heart diseases (like congenital heart disease) with a double number of convolution layers architecture (44). HeartENN is established with two neural network-based epigenomic effects models, one for predicting heart-specific human chromatin features (histone marks, transcription factors and DNase I accessibility) and the other for mice. To assess the utility of the HeartENN model, developers applied it to the WGS data from 749 CHD trios and 1,611 unaffected trios. They found that variants prioritized by HeartENN damaging score (scores ≥0.1) exhibited significant enrichment of the known human CHD genes in CHD cases. Cooperating with a strategy focused on human fetal cardiac enhancers, they confirmed that genes enriched for noncoding DNVs in human fetal cardiac enhancers also have an excess burden on the noncoding DNVs with HeartENN scores ≥0.1, suggesting the capability of the HeartENN in the prioritization of potentially disruptive regulatory noncoding DNVs implicated in CHD (44).

Multiscale Analysis of Regulatory Variants on the Epigenomic Landscape (MARVEL) is developed with a ML algorithm GLM-LARS (generalized linear model-based least angle regression) to prioritize phenotype-associated noncoding variants using WGS data and cell-type specific epigenomic profiles. It integrates gene annotation information, publicly available epigenetic data (e.g., enhancers, promoters, transcription factor motifs) from relevant tissues and the covariates of sample phenotypes to identify potential regulatory regions affected by the noncoding variants. The developers applied MARVEL to the WGS data of 431 short-segment Hirschsprung disease (S-HSCR) cases and 487 ethnically matched controls. Together with ChIP-seq and ATAC-seq data of the human pluripotent stem cell (hPSC)-derived enteric NC-like cells (hNC), they uncovered multiple novel genes implicated in S-HSCR by affecting neural crest migration and development (45).

## 4. Current advances and challenges in variant interpretation

While AI-based tools have made significant contributions to the detection and prioritization of disease-causing variants (Table 1), a persistent challenge in genomic research lies in variant interpretation. In 2015, the ACMG/AMP published an authoritative guideline to standardize variant interpretation, which categorizes variants into five classes ranging from benign to pathogenic (30). Subsequently, multiple platforms were developed for automated variant interpretation based on the ACMG/AMP criteria, such as VarSome, VSClinical, and AION from Nostos genomics. However, although these platforms have facilitated the effective and efficient prioritization of pathogenic or likely pathogenic variants along with their supporting evidence, they still face challenges in interpreting variants of uncertain significance (VUS).

**Table 1:** AI-based tools utilized in the detection and prioritization of disease-causative variants.

Purpose	Variant type	Tools	Methods	Launch year
Variant calling	SNVs/indels	GATK	HMM, Bayesian, etc.	2010
		FreeBayes	Bayesian	2012
		DeepVariants	Deep CNN	2018
	CNVs	PennCNV	HMM	2007
		CN-Learn	Random Forest	2019
		CNV-JACG	Random Forest	2020
		DeepCNV	Deep CNN	2021
Variant prioritizing	Coding variants	CADD	SVM	2014
		REVEL	Random Forest	2016
	Splicing variants	SpliceAI	Deep CNN	2019
		MMSplice	Deep CNN	2019
	Regulatory variants	DeepSEA	Deep CNN	2015
		HeartENN	Deep CNN	2020
		MARVEL	GLM-LARS	2020

HMM, hidden markov model; CNN, convolutional neural networks; SVM, support vector machine; GLM-LARS, generalized linear model-based least angle regression.

## 5. Conclusion

**Figure 2:**
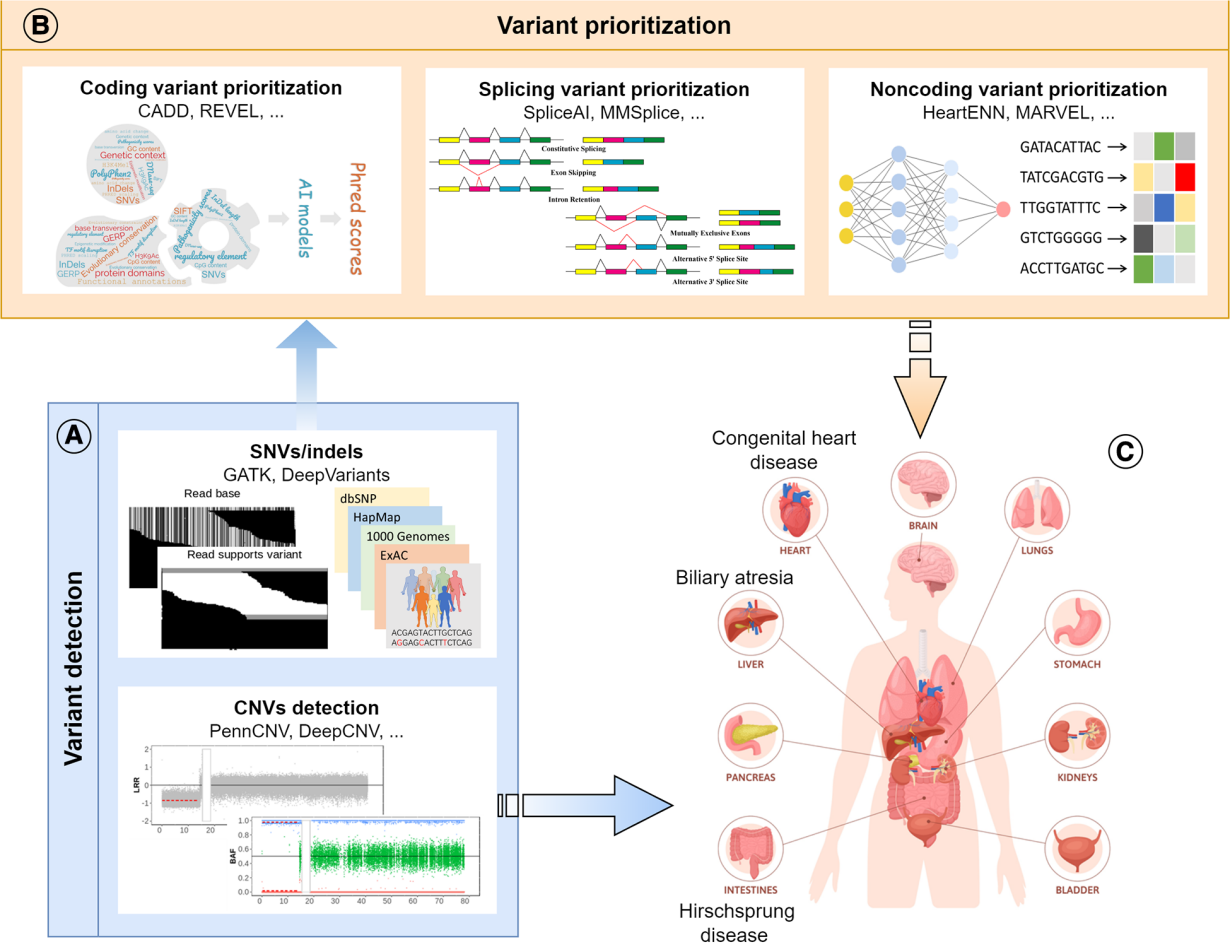
Schematic diagram of the review. (**A**) Application of AI models in variant detection, including SNVs, indels and CNVs; (**B**) Application of AI models in the prioritization of disease-causing variants in different genomic regions (coding, splicing, and noncoding); (**C**) Application of AI models in the research of congenital surgical diseases.

AI makes it possible to integrate and model vast amounts of genomic data quickly and accurately, facilitating the identification, annotation and prioritization of genetic mutations that contribute to disease development (Figure 2). However, large amounts of diverse data are required to train the AI models. Small sample sizes and the lack of diversity in the data available for genomic analysis can limit the accuracy and reliability of the results generated by these models. Moreover, due to the potential variability in predicted outputs generated by distinct AI models, clinicians and researchers may encounter difficulties discerning the most precise outcome and interpreting the underlying pathomechanisms of congenital diseases. Overall, AI has been confirmed to be a powerful tool that revolutionizes disease-specific genomic analysis by providing speedy and precise insights into the complex relationship between genetics and disease development. Ultimately, these findings that traditional methods might have missed will lead to earlier diagnosis and better prognoses for patients with complex congenital disorders.
